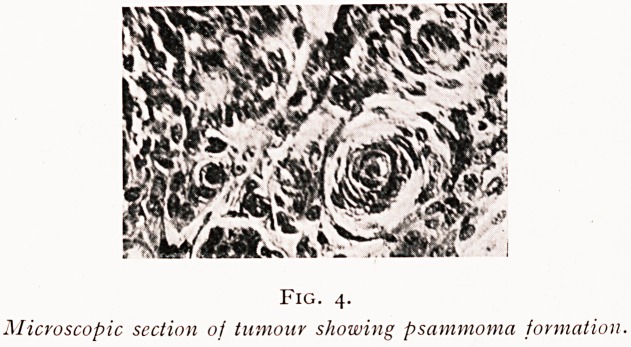# A Case of Intrathecal Extramedullary Spinal Tumour

**Published:** 1925

**Authors:** A. Wilfrid Adams

**Affiliations:** Assistant Surgeon, Bristol Royal Infirmary


A CASE OF

### INTRATHECAL EXTRAMEDULLARY SPINAL TUMOUR—

### CONFIRMATORY LOCALISATION BY LIPIODOL,[1] RELIEF BY OPERATION.

BY

A. Wilfrid Adams, M.S., F.R.C.S.,
*Assistant Surgeon, Bristol Royal Infirmary.*

The interest of this case is twofold.

Firstly, it demonstrates the use of the intrathecal injection of lipiodol as a precise means of localising compression of the spinal cord, and what becomes of the solution.

Secondly, only one other spinal tumour is recorded in the index of surgical cases in the Bristol Royal Infirmary for the past fifteen years.

H.W., aged 64 years, first suffered with varicose veins of the legs twenty months previously, and three months later pain and stiffness started in the knees, especially in the right leg, which began to drag on walking. There were also pains around the lower abdomen. Examination revealed nothing beyond spasticity of the legs. He had noticed the legs draw up involuntarily when lying in bed.

About four months before the operation Dr. Philip T. Jones wrote saying that the patient's condition was deteriorating rather rapidly; large trophic sores had developed over the sacrum, left trochanter and heels; œdema of the legs and lower part of the back appeared, and sphincteric control was lost.

February, 1925. — Examination preceding operation: Condition of organs generally healthy, although he had been unable to stand for fourteen months.

[1] A heavy inert solution of iodine (40 per cent.) in vegetable oil, which is opaque to the X-rays.

The nervous system showed a perfect picture of complete anæsthesia and spastic paraplegia from the toes to the level of the epigastrium. A striking feature was the mass response of the leg muscles on a slight stimulus, such as eliciting Babinski's sign, when both limbs were flexed up at ankle, knee and hip.

*Diagnosis.*—The cause of the symptoms did not appear to be due to primary chronic disease of the cord. Disseminated sclerosis was excluded by age of onset, amyotrophic lateral sclerosis by absence of atrophy in the muscles of the hand, and there was no reason to suspect a combined degeneration of the cord of toxic origin. There was no evidence of injury or disease of the vertebral column which might be compressing the cord, and the radiogram was normal. Wassermann's reaction was negative.

The conclusion was that a tumour within the vertebral canal was the cause of pressure on the cord about at the level of its eighth dorsal segment, and this was confirmed by :—

(*a*) Cerebrospinal fluid withdrawn from the cisterna magna being clear and colourless, while Dr. A. D. Fraser reported that what was drawn from the lumbar region showed " cells 8 per cm. (mononuclear), albumen 0.3 per cent., glucose 0.03 per cent. Fluid is yellow, and a network has formed (Froin's syndrome positive)."

(*b*) Injection of lipiodol and radiography.

On June 2nd, with the patient supported in the sitting position, under local anæsthesia, a lumbar puncture needle was inserted about one inch below the inion and guided along the occipital bone to the foramen magnum. Drops of cerebro-spinal fluid appeared, and 1 cc. of lipiodol was injected. (The injection troubled the patient even less than the lumbar puncture.) Skiagraphy showed the descent of the fluid in the theca was arrested completely at the level of the sixth dorsal vertebra, and continued to be arrested there ninety-six hours later! (Fig. 1.) The inference from this was that the fluid had settled on the upper limit of the tumour where this was compressing the cord and blocking the subarachnoid space. With this visible evidence to confirm the clinical data the diagnosis was made of intrathecal spinal tumour at the level of the eighth–ninth dorsal segments,

Fig. 1.

*Before removal of tumour showing lipiodol arrested at level of 6th dorsal vertebra.*

Fig. 2.

*After removal, showing lipiodol at level of 5th lumbar vertebra.*

and as such the patient was shown before the meeting of the Society on February 11th.

*Operation.*—February 17th. Removal of the tumour.

The dura was exposed by raising the laminæ from the fifth to ninth dorsal vertebræ. No abnormality was visible. but at about the middle of the length of exposed theca an area of increased firmness was felt, and corresponded to the site suggested by the lipiodol shadow, and then incision of the dura and arachnoid over the suspicious region brought the tumour into view, about one inch in length, purplish in colour and slightly adherent. It came out in two fragments as shown in Fig. 3, proving the accuracy of lipiodol as a localiser in obstruction of the spinal theca. Dr. A. D. Fraser kindly reported on the microscopic appearance as "soft psammoma" (Fig. 4). The well-marked bed in the

FIG. 3.
*Tumour in two portions, natural size.*

cord formerly occupied by the tumour appeared to fill out but little. The dura was closed carefully and muscles brought together over it. Primary union occurred in the wound.

*Subsequent course.*—The stiffness of the limbs is much reduced. Voluntary movements are present, though still weak. With assistance he first stood for a minute on the tenth week. The sacral sore is improved but troublesome still, while the others are healed or nearly so ; œdema has gone from the legs and they are warmer, and sphincter control has returned in the bladder. There is some slight return of sensation, and pain has been felt in the sore on the left hip.

FIG. 4.
*Microscopic section of tumour showing psammoma formation.*

*Prognosis.*—The extent of his ultimate recovery is difficult to foretell. It is now three months since operation, and he is still slowly improving.

What becomes of the lipiodol was shown in an interesting way by the radiogram taken three months after operation (Fig. 2). Since the removal of the obstructing tumour it had trickled down to the level of the fifth lumbar vertebra. Very little absorption seems to have occurred, which points to the inert nature of the injected lipiodol.

## Figures and Tables

**Fig. 1. f1:**
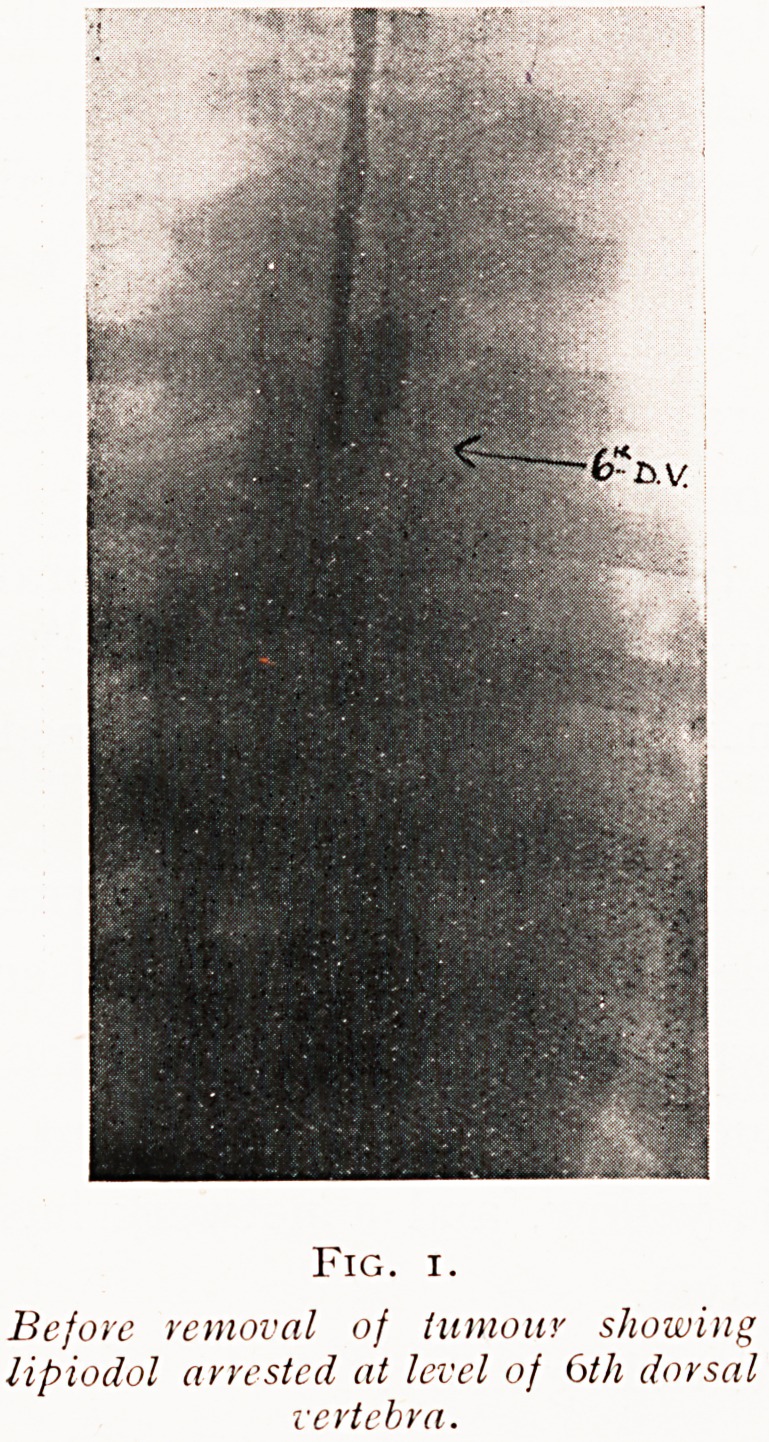


**Fig. 2. f2:**
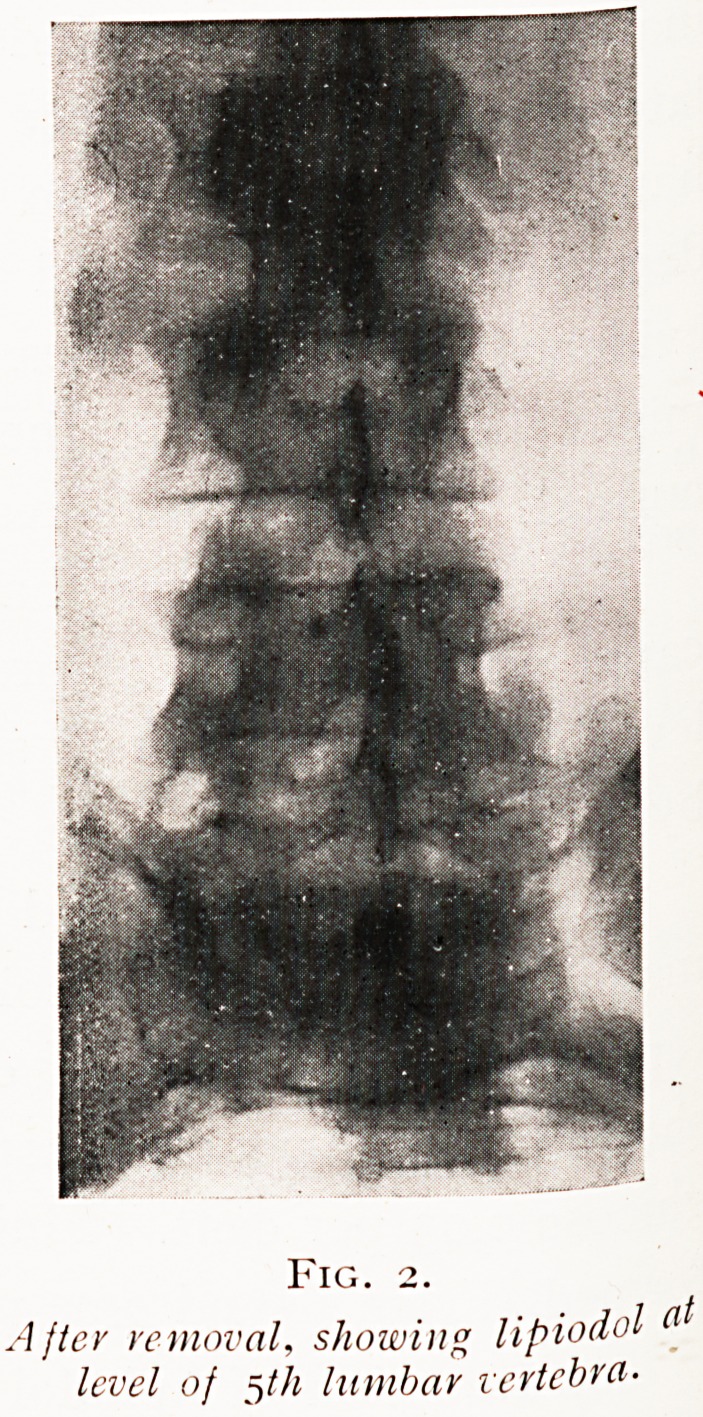


**Fig. 3. f3:**
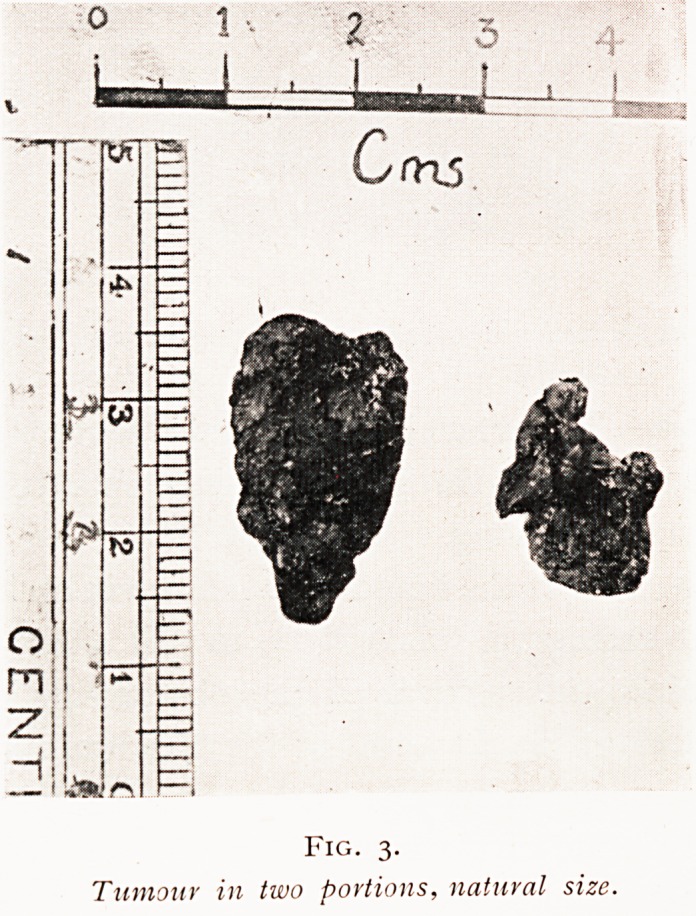


**Fig. 4. f4:**